# Supplementary material for: Principles of coarse-scale functional organization in occipitotemporal cortex
Source: Commun Biol. 2026 Nov 7;9:1483. doi: 10.1038/s42003-026-11319-0 (PMC13744571; doi:10.1038/s42003-026-11319-0)
Supplement: Supplementary file 1 [file 42003_2026_11319_MOESM1_ESM.pdf]

Supporting information for

Principles of coarse-scale functional organization in occipitotemporal cortex

Laura M. Stoinski, Talia Konkle, & Martin N. Hebart

Laura M. Stoinski
Email: stoinski@cbs.mpg.de

This PDF file includes:

Supplementary Figure 1–4
Supplementary Table 1–3
Supplementary Methods and Results
Supplementary References

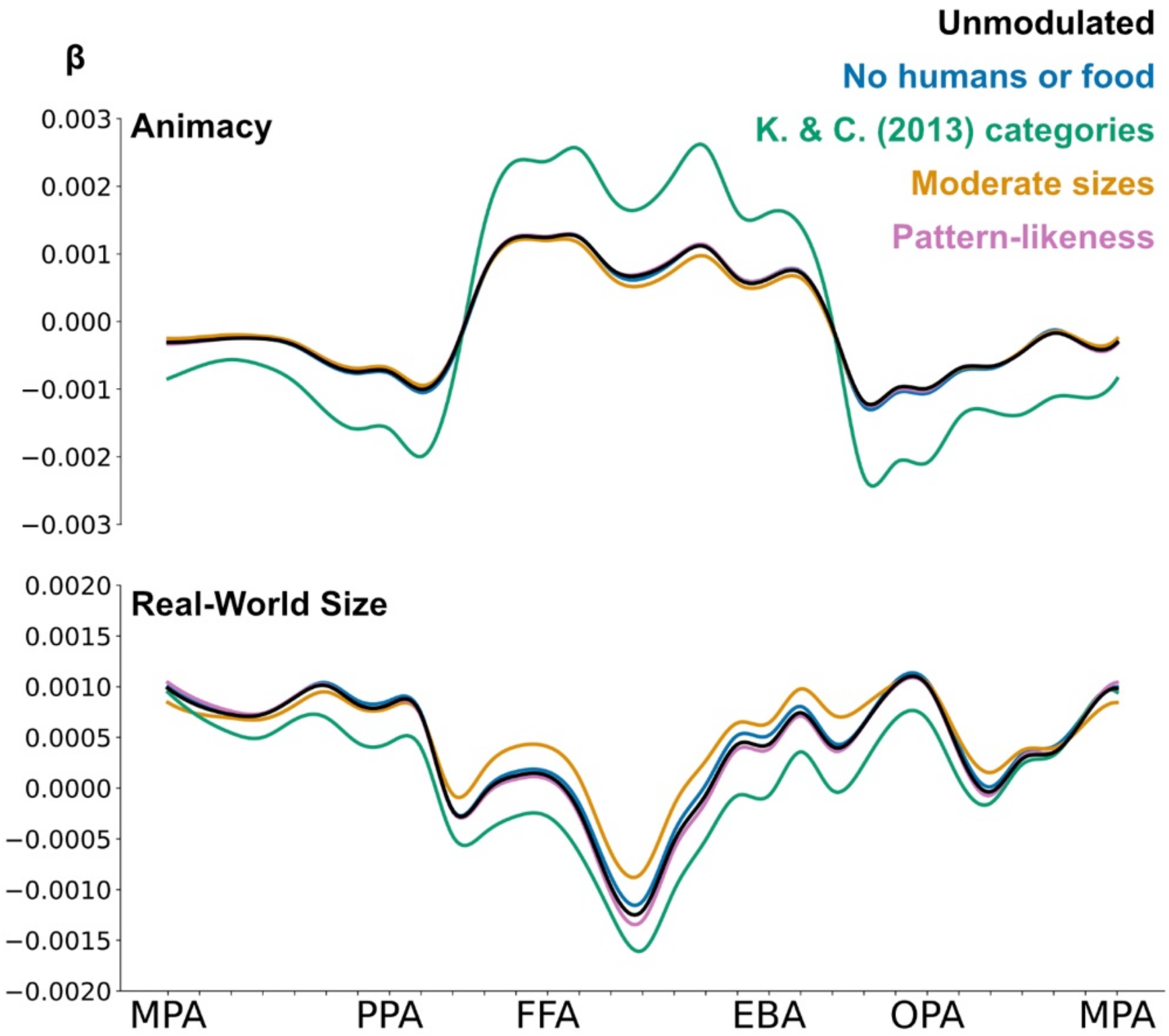

Supplementary Fig 1. Robustness of animacy and size response profiles to exploratory control analyses. To assess the stability of the observed animacy and real-world size organization, we recomputed response profiles in high-level visual cortex after systematically modifying the stimulus set. Profiles are averaged across the three participants. Control analyses were designed to approximate the stimulus set of the original study and to test potential stimulus-driven confounds. Specifically, we excluded images containing humans or food, restricted analyses to the object categories used in Konkle & Caramazza[1], removed categories with extremely small or large real-world sizes, and excluded images with highly pattern-like structure. Across these modulations, the finer-grained size response patterns remained similar, indicating that the reported organization is unlikely to be driven by these stimulus factors.

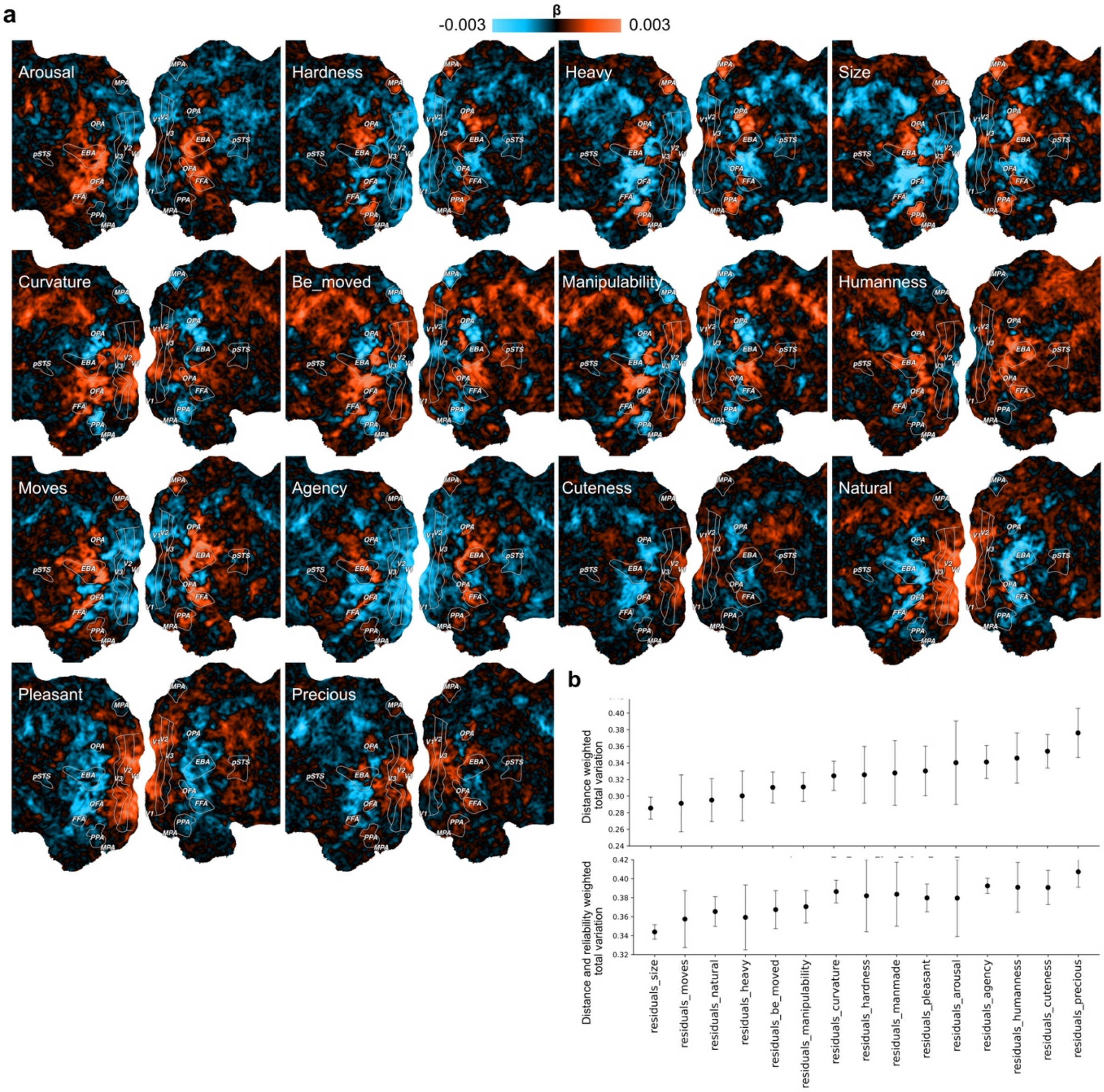

Supplementary Fig 2. Smoothness of topographic property maps after controlling for animacy. a. Topographic maps of object properties after regressing animacy-related variance out of property scores and fMRI responses. **b.** Corresponding smoothness scores for the residualized property topographies. Many properties continued to exhibit broad and spatially smooth topographies after controlling for animacy, indicating that their smoothness cannot be explained solely by variance shared with animacy**.**

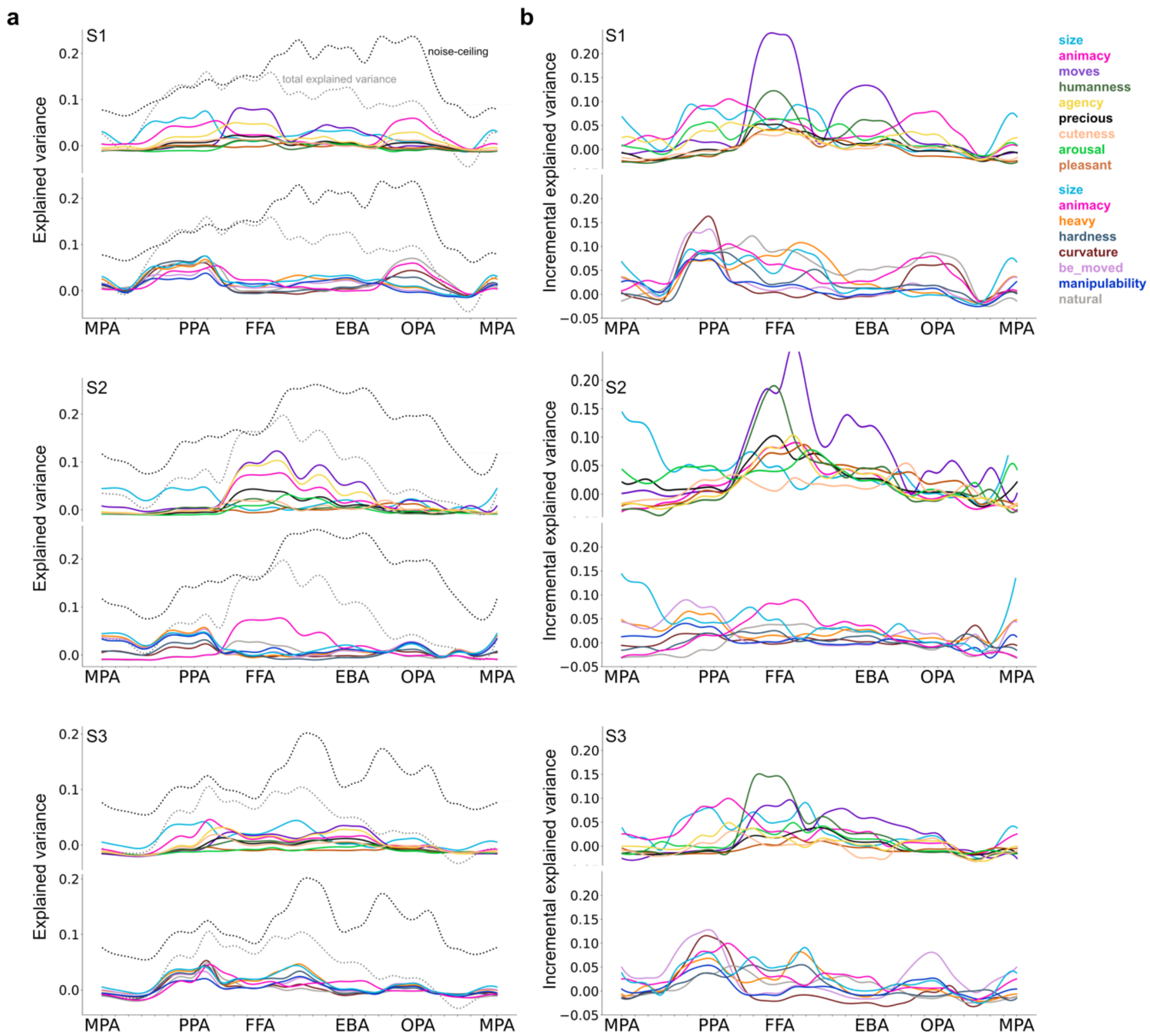

Supplementary Fig 3. Participant-wise explained variance by object property across high-level visual cortex. a. Noise ceiling of the fMRI data, and total variance explained by univariate models for each property regressor and full model including all properties (cross validated). Across participants, moveability, agency, animacy, and size explained the largest shares of variance overall. **b.** Incremental variance explained by each property, estimated using hierarchical partitioning (cross-validated and noise-normalized). Across participants, moveability and humanness consistently explained most incremental variance in FFA and EBA. In other regions, incremental variance was more evenly distributed across properties, and the ranking of properties explaining the most incremental variance varied slightly more across participants.

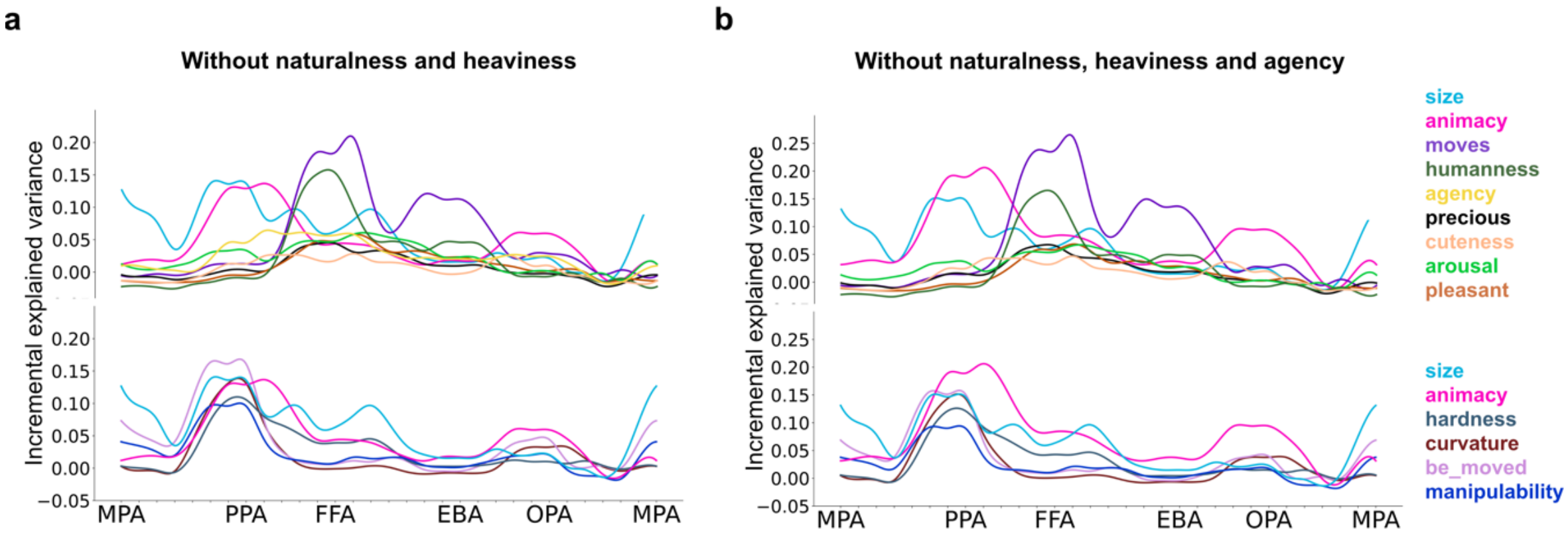

Supplementary Fig 4. Effects of modifying the set of predictors on the hierarchical partitioning analysis. a. Hierarchical partitioning results after excluding naturalness and heaviness. The resulting pattern was highly similar to the full analysis, with moveability and humanness explaining most incremental variance in FFA and EBA. **b.** Hierarchical partitioning results after additionally excluding agency. The overall pattern remained similar to the full analysis, with moveability and humanness explaining most incremental variance in FFA and EBA.

Supplementary Table 1. Object concepts eliciting the highest and lowest responses in a selection of spherical ROIs.

Sphere	7 (PPA)	8	12 (FFA)	15	19 (EBA)	24 (OPA)	30 (MPA)
Highest response in ROI	subway	waterfountain	chest1	hip	hip	submarine	submarine
	desk	bumper	hip	chest1	pogo_stick	hammock	bed
	tent	desk	girl	lingerie	footbath	pothole	hammock
	bumper	subway	woman	helmet	chest1	pogo_stick	waterfountain
	submarine	pothole	pogo_stick	garter	leggings	airbag	treadmill
	waterfountain	submarine	face	tadpole	iceskate	waterfountain	carousel
	pothole	tent	shower_cap	snorkel	snorkel	footbath	pogo_stick
	railing	mosquito_net	leggings	leggings	footrest	tent	clay
	stalagmite	footbath	garter	octopus	anklet	credit_card	tablecloth
	mosquito_net	hammock	hamster	tattoo	dog	desk	eyepiece
	hammock	guillotine	snorkel	wooden_leg	rollerblade	helmet	banana
	sodafountain	bed	monkey	anklet	kimono	hip	subway
	solar_panel	meat_grinder	dog	pacifier	thumbtack	guillotine	manatee
	seesaw	dishwasher	kimono	retainer	hamster	computer_screen	snowboard
	meat_grinder	wooden_leg	doll	prism	snake	subway	doormat
Lowest response in ROI	pumpkin	bungee	kettle	cage	radiator	lettuce	bag
	bear	kettle	radiator	beaker	croissant	bag	cinnamon
	squirrel	squirrel	cup	fire	altar	peanut	rabbit
	potato	peanut	lime	dollhouse	fern	steak	bandage
	lime	sheep	fence	christmastree	manhole	tray	uniform
	cup	rabbit	parsley	tree	fence	cookie	nacho
	stethoscope	cup	cherry	speaker	jar	lime	record
	macadamia	stethoscope	speaker	poster	beaker	possum	hammer
	cashew	uniform	fern	cherry	scaffolding	croissant	cufflink
	croissant	potato	scaffolding	altar	speaker	sheep	stethoscope
	cherry	hand	cage	fence	cage	macadamia	blowtorch
	hand	cherry	altar	parsley	couch	parsley	elbow
	rhinoceros	croissant	towel_rack	scaffolding	cherry	breadstick	butterfly
	kettle	rhinoceros	couch	couch	parsley	bamboo	mousse
	parsley	parsley	bamboo	bamboo	bamboo	cherry	tray

Supplementary Table 2. Object concepts with the lowest and highest residuals in spherical ROI 12 (FFA) for selected property-based encoding models

Model:	Animacy	Size	Moves	Humanness	Agency
Lowest absolute residuals	burner	corn	cauliflower	subway	pocketknife
	chocolate	egg	blanket	velcro	penguin
	mosquito	rocking_chair	lasagna	egg	syringe
	sleeping_bag	wheelbarrow	possum	eyepiece	joystick
	bee	snowplow	icicle	granite	playing_card
	groundhog	space_shuttle	bear	thimble	ping-pong_table
	anchor	mop	celery	broom	porcupine
	donut	pie	crutch	corn	hedgehog
	birdcage	missile	chalk	camcorder	frog
	nest	ping-pong_table	chalice	quill	doormat
	hovercraft	bird	candelabra	crayon	pecan
	popsicle	pothole	hanger	jersey	meatball
	zebra	icicle	bed	needle	corkscrew
	trap	crayon	soda_fountain	hatbox	snowplow
	mosquito_net	oyster	fish	donkey	treadmill
Highest positive residuals	hip	chest1	chest1	chest1	hip
	chest1	hip	hip	pogo_stick	chest1
	pogo_stick	girl	shower_cap	hip	pogo_stick
	shower_cap	woman	garter	otter	shower_cap
	leggings	pogo_stick	leggings	snorkel	leggings
	garter	shower_cap	snorkel	wolf	snorkel
	snorkel	leggings	doll	shower_cap	hood
	doll	garter	pogo_stick	dog	kimono
	girl	hamster	kimono	girl	garter
	kimono	snorkel	girl	lobster	girl
	woman	monkey	tattoo	tadpole	anklet
	hood	dog	helmet	hamster	woman
	robot	doll	woman	platypus	doll
	helmet	kimono	hood	woman	chin
	tattoo	wolf	footbath	warthog	footbath
Highest negative residuals	bamboo	bamboo	bamboo	bamboo	bamboo
	parsley	couch	bus	couch	wheat
	wheat	altar	couch	cherry	parsley
	cherry	scaffolding	tractor	scaffolding	cherry
	couch	cage	fire	kettle	couch
	lime	speaker	wheat	altar	altar
	hedge	parsley	golf_cart	lime	bus
	pumpkin	cherry	aircraft_carrier	speaker	fire
	altar	fence	parsley	parsley	speaker
	scaffolding	lime	cage	cage	giraffe
	cage	radiator	giraffe	fence	cage
	grape	wheat	scaffolding	hairpin	lime
	flower	tray	wheelchair	bus	radiator
	peanut	kettle	mast	dresser	hedge
	speaker	bus	bike	croissant	scaffolding

Supplementary Table 3. Object concepts with the lowest and highest residuals in spherical ROI 19 (EBA) for selected property-based encoding models

Model:	Animacy	Size	Moves	Humanness	Agency
Lowest absolute residuals	spatula	goalpost	filing_cabinet	undershirt	crown
	possum	inhaler	book	comb	life_jacket
	inhaler	pecan	papaya	pear	hanger
	life_jacket	walker1	sword	paint	lemon
	rocking_chair	cow	toothpick	blazer	grate
	garbage	sushi	zucchini	crib	spatula
	blender	polaroid	bench	key	milk
	polaroid	fish	ferris_wheel	cellphone	ruler
	grate	blazer	dishwasher	boy	skunk
	hanger	bulldozer	ruler	blind	boxing_gloves
	tomato	drum	chicken_wire	egg	donut
	antenna	crayon	peeler	antenna	sushi
	bib	net	leek	tiara	spider
	milk	airplane	pan	stair	lollipop
	projector	onion	football_helmet	garbage	raft
Highest positive residuals	pogo_stick	hip	pogo_stick	pogo_stick	pogo_stick
	hip	pogo_stick	hip	hip	hip
	footbath	footbath	footbath	footbath	footbath
	leggings	leggings	leggings	snorkel	iceskate
	iceskate	chest1	snorkel	footrest	footrest
	snorkel	snorkel	footrest	iceskate	anklet
	footrest	iceskate	anklet	thumbtack	leggings
	anklet	anklet	iceskate	snake	snorkel
	chest1	footrest	thumbtack	dog	chest1
	rollerblade	thumbtack	kimono	leggings	rollerblade
	thumbtack	floss	floss	anklet	thumbtack
	kimono	dog	credit_card	bow2	kimono
	floss	hamster	chest1	chest1	bow2
	bow2	rollerblade	brace	dolphin	boot
	boot	snake	boot	rollerblade	wooden_leg
Highest negative residuals	bamboo	bamboo	bamboo	bamboo	bamboo
	parsley	couch	parsley	cherry	parsley
	cherry	cage	cage	parsley	cherry
	couch	parsley	cherry	couch	speaker
	cage	speaker	couch	speaker	cage
	speaker	cherry	speaker	cage	couch
	scaffolding	scaffolding	tractor	scaffolding	beaker
	beaker	fence	bus	croissant	scaffolding
	jar	beaker	scaffolding	jar	jar
	asparagus	jar	beaker	nacho	altar
	fence	altar	rhinoceros	fence	radiator
	croissant	radiator	golf_cart	beaker	fence
	tree_trunk	dollhouse	aircraft_carrier	bottle	asparagus
	altar	croissant	banner	altar	croissant
	potato	rug	jar	dresser	bottle

Supplementary Methods and Results

Robustness of full factorial organization of animacy and size to stimulus effects

We conducted various exploratory analyses to evaluate the robustness of our animacy and size topographies (Supplementary Fig. 1). First, we confirmed that the additional size preference regions were also visible in the volumetric data, ensuring they were not a result of erroneous surface mapping. Second, we ensured that the size topography remained consistent when neglecting the continuity of our size measure, only comparing sensitivities to the 25% largest and the 25% smallest categories, similar to how it was done in the original work[1].

Further, we investigated the influence of category diversity. The original study[1] did not include images of humans or food. In light of the strong response preferences of FFA and EBA to human faces and bodies[2,3] and the recently proposed food-selective region between FFA and PPA[4,5], we tested the influence of these categories on our results. However, excluding images of food, human faces and bodies, or even repeating the analysis only with objects included in the original study (n = 101), did not alter the more fine-grained size partition. Animate and inanimate categories covered largely overlapping size ranges with only ten objects falling outside the range spanned by animals, which extended from "ant" to "whale". However, compared to the original work[1], our categories varied more strongly in their size, ranging from a small grain of sand up to an aircraft carrier. To investigate the impact of size range, we repeated the analysis excluding extremely large (size > 320) and extremely small (size < 140) categories (excluded n = 84). We also compared response to only the 25% largest and smallest categories. Yet, we observed a similar pattern of responses, indicating that this effect is not solely due to the wider range and continuity of our size ratings.

We additionally explored whether characteristics other than size might explain the additional size regions in our data. Size was only weakly correlated with the eccentricity (r = 0.02, p = 0.16) or the size at which the objects are displayed in the images (r = 0.06, p < 0.001).

We compared animacy and size ratings with image-wise estimates of other visual dimensions, as determined from human similarity judgments of the THINGS images[6–8]. Animacy and size were only weakly correlated with color-related dimensions (all $| r | < 0.16$). However, size correlated with the "coarse-scale pattern/many things" dimension ($r = -0.49$, $p < 0.001$), as smaller categories were more frequently portrayed collectively in pattern-like arrangements (e.g., a pile of lentils). However, excluding fine-grained pattern-like images (i.e., pattern-likeness > 0.6, excluded $n = 354$) before averaging the responses across images showing the same concept did not alter the observed animacy and size response profiles.

Finally, the original studies[1,9] may have overlooked finer size partitions as they assessed size topography in group averages rather than individual participants, as done here, potentially obscuring these details in the spatially normalized and smoothed results. However, even after testing the effects of averaging and applying a broad smoothing kernel (FWHM = 6–8 mm), we continued to observe a finer size partition.

Smoothness of property topographies after controlling for animacy

To explore whether the smoothness of properties correlated with animacy merely reflects variance shared with animacy, we regressed animacy-related variance out of both the property scores and the fMRI responses and then computed regression slope maps for the residualized object properties. Smoothness was quantified using the same procedure described in the Smoothness of property topographies analysis in the Methods section of the main manuscript, with the exception that we used 10 bootstrap iterations instead of 1,000 to reduce computational demands for this exploratory analysis. Supplementary Fig. 2a shows the resulting topographical maps after controlling for animacy, and Supplementary Fig. 2b shows the corresponding smoothness scores. Even after removing variance associated with animacy, many properties continued to produce broad and spatially smooth maps, suggesting

that their topographical smoothness cannot be explained solely by their correlation with animacy.

Participant-wise differences in hierarchical partitioning results

To assess the consistency of property effects across participants, we plotted the results shown in Fig. 7 of the main manuscript separately for each participant. Supplementary Fig. 3a shows the variance explained by each property, estimated using univariate OLS regression models. Supplementary Fig. 3b shows the incremental variance explained by each property, estimated using hierarchical partitioning.

Overall, participant-wise patterns were broadly consistent with the group average. Moveability and humanness consistently remained the properties explaining most incremental variance in FFA and EBA. In other regions, the ranking of the properties explaining most incremental variance varied slightly more across participants. In PPA, curvature and the ability to be moved explained most incremental variance in S1; the ability to be moved and heaviness in S2; and the ability to be moved and curvature in S3. In the previously proposed small-object region between FFA and EBA[1], size and heaviness explained the most incremental variance in S1 and S3, whereas moveability explained most incremental variance in S2. In OPA, naturalness and animacy explained most incremental variance in S1; moveability in S2; and the ability to be moved in S3. Together, these results demonstrate that the pronounced effects of moveability and humanness in FFA and EBA, as well as the more balanced contribution of multiple properties to explaining responses in other regions, are consistent across participants.

Robustness of hierarchical partitioning results to modulations of the predictor set

Because many of the investigated properties were correlated with animacy or size, we further examined whether excluding highly correlated properties affected the hierarchical partitioning

results (for correlations, see Fig. 4a of the main manuscript). We first repeated the analysis without naturalness and heaviness, which were strongly correlated with animacy or size, respectively. We then additionally excluded agency because of its high correlation with animacy.

As shown in Supplementary Fig. 4, both analyses yielded highly similar results. As expected, including fewer predictors slightly increased the incremental variance explained by the remaining predictors. This was particularly the case for animacy, size, and the ability to be moved in PPA and OPA. As in the full analysis (Fig. 7c of the main manuscript), moveability and humanness explained more incremental variance than animacy in FFA and EBA, and size, animacy, and the ability to be moved explained the most incremental variance in PPA. In OPA, however, animacy explained the most incremental variance after excluding correlated predictors, whereas the full analysis showed a more balanced contribution of animacy, size, and the ability to be moved. Overall, these results show that the strong effects of moveability and humanness beyond animacy are robust and do not simply reflect a bias of including multiple animacy-related properties in the full property set.

Exploration of objects eliciting the highest and lowest responses within spherical ROIs.
To identify the object concepts eliciting the highest and lowest responses in each spherical ROI, we averaged the single-trial beta estimates provided by the THINGS-fMRI dataset[7] across the 12 images depicting each object concept, and then averaged these object-wise responses across participants. Supplementary Table 1 shows the 15 objects eliciting the highest responses and the 15 objects eliciting the lowest responses within a subset of ROIs overlapping with animacy- or size-preference regions identified in the present study (see Fig. 2 of the main manuscript).

The resulting profiles broadly matched known animacy-, size-, and category-selective organization in occipitotemporal cortex. ROIs previously related to large-object and scene preferences, including PPA, MPA, and OPA[1,10], showed stronger responses to large inanimate objects, such as subway, submarine, and carousel, and weaker responses to animate or natural categories, such as bear and rabbit, as well as smaller inanimate objects, such as cup and blowtorch. In animacy-, face-, and body-selective ROIs, including FFA and EBA[1–3], the strongest responses were elicited by animate concepts, body parts, people, animals, and categories often depicted with bodies, such as clothing, whereas the weakest responses were predominantly elicited by inanimate objects.

However, there were exceptions to this pattern. For example, small inanimate objects such as credit cards elicited relatively strong responses in OPA, and body-related concepts such as hip also appeared among higher-ranking concepts. Thus, the ROI response profiles broadly followed known animacy-, size-, and category-preference gradients, while also showing concept-specific deviations not captured by broad labels such as animate, inanimate, large, or small.

Exploration of objects with lowest or highest prediction errors within spherical ROIs based on property encoding models

To explore which objects contributed to the larger shares of explained variance for moveability and humanness compared to animacy in FFA and EBA (Fig. 7b and c of the main manuscript), we examined object-wise prediction errors from the corresponding univariate encoding models of these properties. We additionally included the prediction errors of the size and agency encoding models, as these models also explained substantial variance in these regions.

Supplementary Tables 2 and 3 list the objects with the 15 lowest absolute, the 15 highest positive and the 15 highest negative residuals in FFA and EBA, respectively. Residuals

were defined as measured minus predicted fMRI responses. Low absolute residuals thus indicate objects whose fMRI responses were well predicted by the model. High positive residuals indicate objects for which the model underestimated the measured response, whereas negative residuals indicate objects for which the model overestimated the measured response.

In both FFA (Supplementary Table 2) and EBA (Supplementary Table 3), objects with the largest positive residuals included several clothing categories and body parts. These categories may receive relatively low animacy, moveability, agency, or humanness ratings despite depicting or strongly resembling aspects of the human body. For the animacy model, responses to plants, which received relatively high animacy ratings among non-animal categories, were frequently overestimated. For the moveability model, vehicles were among the objects whose responses were overestimated. Thus, the residual patterns provide additional insight into which object categories are not well captured by individual predictors and suggest that the higher contribution of moveability than animacy in FFA and EBA is unlikely to simply reflect a general preference for both animals and highly moveable inanimate objects such as vehicles.

Supplementary References

1. Konkle, T. & Caramazza, A. Tripartite Organization of the Ventral Stream by Animacy and Object Size. *J. Neurosci.* **33**, 10235–10242 (2013).
2. Downing, P. E., Jiang, Y., Shuman, M. & Kanwisher, N. A Cortical Area Selective for Visual Processing of the Human Body. *Science* **293**, 2470–2473 (2001).
3. Kanwisher, N. & Yovel, G. The fusiform face area: a cortical region specialized for the perception of faces. *Phil. Trans. R. Soc. B* **361**, 2109–2128 (2006).
4. Jain, N. *et al.* Selectivity for food in human ventral visual cortex. *Commun Biol* **6**, 175 (2023).
5. Khosla, M., Ratan Murty, N. A. & Kanwisher, N. A highly selective response to food in human visual cortex revealed by hypothesis-free voxel decomposition. *Current Biology* **32**, 4159-4171.e9. (2022).
6. Hebart, M. N., Zheng, C. Y., Pereira, F. & Baker, C. I. Revealing the multidimensional mental representations of natural objects underlying human similarity judgements. *Nat Hum Behav* **4**, 1173–1185 (2020).
7. Hebart, M. N. *et al.* THINGS-data, a multimodal collection of large-scale datasets for investigating object representations in human brain and behavior. *eLife* **12**, e82580 (2023).
8. Contier, O., Baker, C. I. & Hebart, M. N. Distributed representations of behaviour-derived object dimensions in the human visual system. *Nat Hum Behav. **8**,* 2179–2193 (2024) doi:10.1038/s41562-024-01980-y.
9. Long, B., Yu, C.-P. & Konkle, T. Mid-level visual features underlie the high-level categorical organization of the ventral stream. *Proc. Natl. Acad. Sci. U.S.A.* **115**, E9015–E9024 (2018).
10. Epstein, R. A. & Baker, C. I. Scene Perception in the Human Brain. *Annu Rev Vis Sci* **5**, 373–397 (2019).
